# COVID-19–Associated Hospitalizations Among U.S. Infants Aged <6 Months — COVID-NET, 13 States, June 2021–August 2022

**DOI:** 10.15585/mmwr.mm7145a3

**Published:** 2022-11-11

**Authors:** Sarah Hamid, Kate Woodworth, Huong Pham, Jennifer Milucky, Shua J. Chai, Breanna Kawasaki, Kimberly Yousey-Hindes, Evan J. Anderson, Justin Henderson, Ruth Lynfield, Francesca Pacheco, Grant Barney, Nancy M. Bennett, Eli Shiltz, Melissa Sutton, H. Keipp Talbot, Andrea Price, Fiona P. Havers, Christopher A. Taylor, COVID-NET Surveillance Team, COVID-NET Surveillance Team, Jeremy Roland, Isaac Armistead, Carol Lyons, Kyle Openo, Lauren Leegwater, Erica Mumm, Mark Montoya, Kerianne Engesser, Sophrena Bushey, Nancy Moran, Nasreen Abdullah, Tiffanie Markus, Melanie Crossland

**Affiliations:** ^1^National Center for Immunization and Respiratory Diseases, CDC; ^2^Epidemic Intelligence Service, CDC; ^3^Division of Birth Defects and Infant Disorders, National Center on Birth Defects and Developmental Disabilities, CDC; ^4^California Emerging Infections Program, Oakland, California; ^5^Career Epidemiology Field Officer Program, CDC; ^6^Colorado Department of Public Health and Environment; ^7^Connecticut Emerging Infections Program, Yale School of Public Health, New Haven, Connecticut; ^8^Emory University School of Medicine, Atlanta, Georgia; ^9^Georgia Emerging Infections Program, Georgia Department of Public Health; ^10^Atlanta Veterans Affairs Medical Center, Atlanta, Georgia; ^11^Michigan Department of Health and Human Services; ^12^Minnesota Department of Health; ^13^New Mexico Emerging Infections Program, University of New Mexico, Albuquerque, New Mexico; ^14^New York State Department of Health; ^15^University of Rochester School of Medicine and Dentistry, Rochester, New York; ^16^Ohio Department of Health; ^17^Oregon Health Authority; ^18^Vanderbilt University Medical Center, Nashville, Tennessee; ^19^Salt Lake County Health Department, Salt Lake City, Utah.; California Emerging Infections Program, Oakland, California; Colorado Department of Public Health and Environment; Connecticut Emerging Infections Program, Yale School of Public Health, New Haven, Connecticut; Georgia Emerging Infections Program, Georgia Department of Public Health, Division of Infectious Diseases, School of Medicine, Emory University, Atlanta, Georgia; Michigan Department of Health and Human Services; Minnesota Department of Health; New Mexico Emerging Infections Program, University of New Mexico, Albuquerque, New Mexico; New York State Department of Health; University of Rochester School of Medicine and Dentistry, Rochester, New York; Ohio Department of Health; Public Health Division, Oregon Health Authority; Vanderbilt University Medical Center, Nashville, Tennessee; Salt Lake County Health Department, Salt Lake City, Utah

COVID-19–associated hospitalization rates are highest among adults aged ≥65 years (*1*); however, COVID-19 can and does cause severe and fatal outcomes in children, including infants (*2*,*3*). After the emergence of the SARS-CoV-2 B.1.1.529 (Omicron) BA.1 variant in December 2021, hospitalizations among children aged <5 years, who were ineligible for vaccination, increased more rapidly than did those in other age groups (*4*). On June 18, 2022, CDC recommended COVID-19 vaccination for infants and children aged ≥6 months (*5*). Data from the Coronavirus Disease 2019–Associated Hospitalization Surveillance Network (COVID-NET)* were analyzed to describe changes in the age distribution of COVID-19–associated hospitalizations since the Delta-predominant period (June 20–December 18, 2021)^†^ with a focus on U.S. infants aged <6 months. During the Omicron BA.2/BA.5–predominant periods (December 19, 2021–August 31, 2022), weekly hospitalizations per 100,000 infants aged <6 months increased from a nadir of 2.2 (week ending April 9, 2022) to a peak of 26.0 (week ending July 23, 2022), and the average weekly hospitalization rate among these infants (13.7) was similar to that among adults aged 65–74 years (13.8). However, the prevalence of indicators of severe disease^§^ among hospitalized infants did not increase since the B.1.617.2 (Delta)-predominant period. To help protect infants too young to be vaccinated, prevention should focus on nonpharmaceutical interventions and vaccination of pregnant women, which might provide protection through transplacental transfer of antibodies (*6*).

COVID-NET conducts population-based surveillance for laboratory-confirmed COVID-19–associated hospitalizations among residents of predefined surveillance catchment areas.^¶^ COVID-19–associated hospitalizations are defined as receipt of a positive SARS-CoV-2 molecular or rapid antigen detection test result during hospitalization or during the 14 days preceding hospital admission. Demographic data were collected on all COVID-19–associated hospitalizations in 13 states and used to calculate age-stratified hospitalization rates.** Clinical data (signs and symptoms at admission,^††^ underlying medical conditions, and indicators of severe disease) were available for infants aged <6 months from 12 states.^§§^ Using previously described methods (*4*), clinical data were collected on all infant COVID-NET cases. Because of the surge in hospitalizations during December 2021 and January 2022, some sites abstracted clinical data on a representative sample of hospitalized infants.^¶¶^ A birth hospitalization was defined as the hospitalization during which the infant was born.

This analysis describes weekly COVID-19–associated hospitalization rates (hospitalizations per 100,000 population) and clinical characteristics of infants aged <6 months during June 20, 2021–August 31, 2022, which includes SARS-CoV-2 Delta (June 20–December 18, 2021), Omicron BA.1 (December 19, 2021–March 19, 2022), Omicron BA.2 (March 20–June 18, 2022), and Omicron BA.5 (June 19–August 31, 2022) variant–predominant periods. Hospitalization rates from the pre-Delta period and among all other age groups are included for comparison. Average weekly rates and demographic and clinical characteristics of infants aged <6 months were compared across variant-predominant periods. Unadjusted weekly COVID-19–associated hospitalization rates were calculated by dividing the total number of hospitalized patients by population estimates within each age group for the counties included in the surveillance catchment area.*** Because population estimates are available in 1-year age increments, population denominators for hospitalization rates among infants aged <6 months were calculated as one half the population estimate for infants aged <1 year. Three-week moving averages are presented for visualization purposes. Rate ratios (RRs) and 95% CIs were calculated. Demographic characteristics, clinical outcomes, and severity across SARS-CoV-2 variant-predominant periods were compared; the Omicron BA.2- and BA.5-predominant periods were combined in analyses because of small sample sizes. During the combined Omicron BA.2/BA.5-predominant period, the proportions of hospitalized infants with underlying medical conditions and symptoms on admission by age group (<1 month, 1–3 months, 4–5 months) were estimated. Wilcoxon rank-sum tests and chi-square tests were used to compare medians and proportions, respectively; p-values <0.05 were considered statistically significant. Percentages were weighted to account for the probability of selection for sampled cases, and further adjusted to account for nonresponse (i.e., an incomplete chart review). Data were analyzed using SAS (version 9.4; SAS Institute). This activity was reviewed by CDC and conducted consistent with applicable federal law and CDC policy.^†††^

During the Omicron BA.2/BA.5-predominant period, the weekly hospitalization rate among infants aged <6 months increased elevenfold (95% CI = 4.3–33.3) from a nadir of 2.2 (week ending April 9, 2022) to a peak of 26.0 (week ending July 23, 2022) and began to decline thereafter (Figure). The mean weekly hospitalization rate in this group was higher during the Omicron BA.2/BA.5 period (13.7) than during the Delta period (8.3) (RR = 1.6; 95% CI = 1.4–1.8). Compared with the Delta period, rates were also higher during the BA.2/BA.5 period among infants and children aged 6 months–4 years (RR = 1.9) and adults aged ≥75 years (RR = 1.4) but lower among children and adolescents aged 5–17 years (RR = 0.9), adults aged 18–64 years (RR = 0.5), and adults aged 65–74 years (RR = 0.8). The mean weekly hospitalization rate among infants aged <6 months during the Omicron BA.2/BA.5 period (13.7) was less than that of adults aged ≥75 years (39.4), similar to that of adults aged 65–74 years (13.8) and higher than rates in all other pediatric age groups (2.3 and 0.8 for children aged 6–23 months and 2–4 years, respectively) and in adults aged <65 years (*4*,*6*).

**FIGURE:**
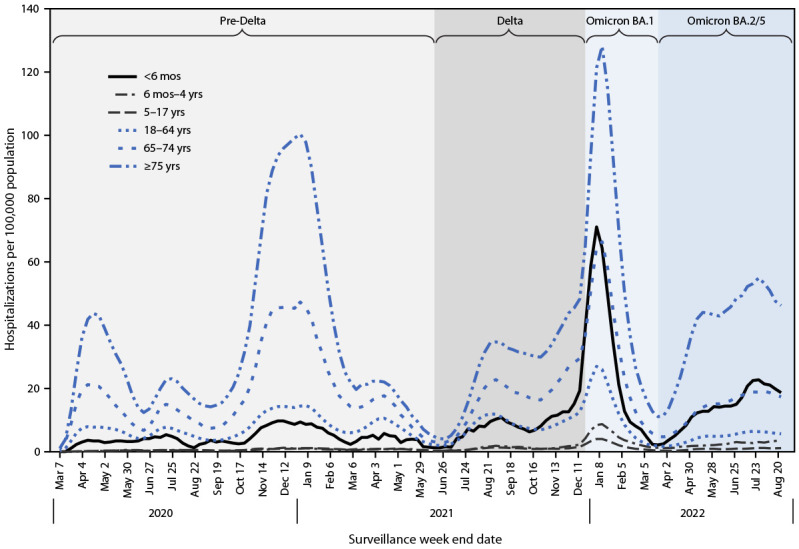
Weekly COVID-19–associated hospitalization rates* by age group (3-week moving average) and period of SARS-CoV-2 variant predominance^†^ — Coronavirus Disease 2019–Associated Hospitalization Surveillance Network, 13 states,^§^ March 2020–August 2022 * Number of patients with a laboratory-confirmed COVID-19–associated hospitalization per 100,000 population. Rates are calculated using the CDC National Center for Health Statistics’ vintage 2020 bridged-race postcensal population estimates for the counties included in the surveillance area (https://www.cdc.gov/nchs/nvss/bridged_race.htm). Rates are subject to change as additional data are reported. ^†^ Periods of SARS-CoV-2 variant predominance are defined as follows: Delta: June 20–December 18, 2021; Omicron BA.1: December 19, 2021–March 19, 2022; Omicron BA.2: March 20–June 18, 2022; and Omicron BA.5: June 19–August 31, 2022. ^§^ Data are collected in selected counties in California, Colorado, Connecticut, Georgia, Maryland, Michigan, Minnesota, New Mexico, New York, Ohio, Oregon, Tennessee, and Utah. A list of these counties is available at https://www.cdc.gov/mmwr/volumes/69/wr/mm6915e3.htm. Additional information on surveillance methods is available at https://www.cdc.gov/coronavirus/2019-ncov/covid-data/covid-net/purpose-methods.html.

Complete clinical data were available for 1,116 infants aged <6 months hospitalized with laboratory-confirmed COVID-19, including 321, 322, and 473 infants hospitalized during the Delta, Omicron BA.1, and Omicron BA.2/BA.5 periods, respectively (Table 1). Differences during the three periods were small, and not all were statistically significant; however, indicators of severity, including length of hospital stay, and the proportion of hospitalizations that required intensive care unit admission, high-flow nasal cannula, mechanical ventilation, or bilevel positive airway pressure/continuous positive airway pressure (BiPAP/CPAP) were consistently lower during the Omicron BA.2/BA.5 period than during the Delta period, and in-hospital deaths were rare (<1%).

**TABLE 1:** Demographic characteristics and clinical outcomes of hospitalized infants aged <6 months with laboratory-confirmed SARS-CoV-2 infections,* by period of SARS-CoV-2 variant predominance — Coronavirus Disease 2019–Associated Hospitalization Surveillance Network, 12 states,^†^ June 20, 2021–August 31, 2022

Characteristic	SARS-CoV-2 variant predominance period, no. (%) of hospitalizations				
	Total	Delta	Omicron BA.1	Omicron BA.2/5	p-value^§^
		(Jun 20–Dec 18, 2021)	(Dec 19, 2021–Mar 19, 2022)	(Mar 20–Aug 31, 2022)	
**Total**	**1,116**	**321**	**322**	**473**	**—**
Age, days, median (IQR)	**46.1 (18.1–100.7)**	41.1 (16.9–83.0)	48.6 (16.8–107.3)	47.5 (20.7–105.6)	0.04
**Sex**					
Male	**625 (56.2)**	168 (52.6)	189 (57.8)	268 (57.0)	0.17
Female	**491 (43.8)**	153 (47.4)	133 (42.2)	205 (43.0)	
**Race and ethnicity^¶^**					
Asian or Pacific Islander, NH	**65 (5.8)**	15 (4.5)	12 (3.4)**	38 (8.4)	0.10
Black or African American, NH	**234 (20.8)**	74 (22.9)	68 (21.3)	92 (19.1)	
Hispanic	**272 (25.0)**	80 (25.4)	79 (25.7)	113 (24.2)	
White, NH	**417 (36.8)**	118 (36.6)	123 (36.7)	176 (36.9)	
All other races**	**30 (2.7)**	9 (2.7)	11 (3.5)	10 (2.1)	
Unknown race or ethnicity	**98 (8.9)**	25 (7.7)	29 (9.3)	44 (9.3)	
**Hospitalization intervention or outcome^††^**					
Length of hospital stay, days, median (IQR)	**1.6 (0.9–2.8)**	1.7 (0.9–3.6)	1.6 (0.9–2.7)	1.5 (0.8–2.6)	<0.01
ICU admission	**234 (20.9)**	72 (22.5)	75 (23.2)	87 (18.0)	0.08
BiPAP/CPAP	**65 (5.7)**	28 (8.7)	16 (4.8)	21 (4.5)	<0.01
High-flow nasal cannula	**157 (13.5)**	51 (15.9)	49 (14.0)	57 (11.6)	0.02
Invasive mechanical ventilation	**53 (5.2)**	17 (5.4)	21 (7.4)	15 (3.2)	<0.01
In-hospital death	**6 (0.7)**	0 (—)	3 (1.3)^§§^	3 (0.6)^§§^	NA

**Abbreviations**: BiPAP/CPAP = bilevel positive airway pressure/continuous positive airway pressure; COVID-NET = Coronavirus Disease 2019–Associated Hospitalization Surveillance Network; ICU = intensive care unit; NA = not applicable; NH = non-Hispanic.

* Data are a weighted sample of hospitalized infants with completed medical record abstractions. Sample sizes presented are unweighted with weighted percentages.

^†^ Includes infants admitted to a hospital with an admission date during June 20, 2021–August 31, 2022, in 12 states participating in COVID-NET: California, Colorado, Connecticut, Georgia, Michigan, Minnesota, New Mexico, New York, Ohio, Oregon, Tennessee, and Utah. https://www.cdc.gov/mmwr/volumes/69/wr/mm6915e3.htm

^§^ Proportions between the Delta variant and Omicron BA.2/BA.5 variant periods of predominance were compared with chi-square tests, and medians were compared with the Wilcoxon rank-sum test, accounting for sampling weights.

^¶^ If ethnicity was unknown, NH ethnicity was assumed.

** Includes NH persons reported as other or multiple races and persons of Alaska Native and American Indian race.

^††^ Hospitalization outcomes are not mutually exclusive.

^§§^ Relative SE >30%.

Among 473 infants aged <6 months hospitalized during the Omicron BA.2/BA.5 variant-predominant period, 397 (84%) had COVID-19–related symptoms. Among all 473 infants, 174 (38%) were aged <1 month; 69 (39%) of these were birth hospitalizations (Table 2). Among infants who received a positive SARS-CoV-2 test result during the birth hospitalization, 60 (87%) were asymptomatic. Excluding birth hospitalizations, similar proportions were hospitalized with COVID-19–related symptoms among infants aged <1 month (94%), 1–2 months (97%) and 3–5 months (96%). At least one underlying medical condition was present in 26% of hospitalized infants aged 1–2 months and 36% of those aged 3–5 months. Prematurity^§§§^ was the most frequently reported underlying condition (20% and 25% of infants aged 1–2 months and 3–5 months, respectively). Most infants aged 1–2 months (74%) and 3–5 months (68%) had fever on admission.

**TABLE 2:** Clinical characteristics of hospitalized infants aged <6 months with laboratory-confirmed SARS-CoV-2 infections during the combined period of SARS-CoV-2 Omicron BA.2 and BA.5 variant predominance* — Coronavirus Disease 2019–Associated Hospitalization Surveillance Network, 12 states,^†^ March 20–August 31, 2022

Characteristic	No. (%) of hospitalizations, by age group			
	All <6 mos	<1 mo	1–2 mos	3–5 mos
**Total no. of hospitalized infants**	**473**	**174**	**154**	**145**
Birth hospitalization^§^	69 (14.8)	69 (39.3)	0 (—)	0 (—)
**Underlying medical conditions**				
≥1 underlying medical condition^¶^	114 (23.5)	20 (11.8)	41 (26.1)	53 (35.6)
Prematurity	86 (17.7)	17 (10.0)	31 (19.6)	38 (25.2)
COVID-19–related symptoms on admission**				
Yes	397 (83.6)	108 (62.2)	149 (96.7)	140 (96.4)
**Symptoms at admission** ^††^				
Fever	287 (60.6)	75 (43.3)	112 (73.5)	100 (68.3)
Congested/Runny nose	220 (45.9)	51 (29.3)	82 (52.7)	87 (59.3)
Cough	218 (45.5)	39 (22.6)	89 (57.4)	90 (61.2)
Inability to eat/Poor feeding	164 (33.9)	32 (18.3)	73 (47.5)	59 (38.9)
Shortness of breath/Respiratory distress	132 (27.6)	27 (15.7)	43 (27.7)	62 (42.5)
Nausea/Vomiting	91 (19.3)	18 (10.9)	36 (23.2)	37 (25.6)
Diarrhea	57 (12.2)	10 (5.8)	19 (12.5)	28 (19.8)
Lethargy	45 (9.5)	11 (6.2)	20 (13.5)	14 (9.2)
Rash	32 (6.6)	6 (3.5)^§§^	18 (11.2)	8 (5.4)^§§^
Apnea	31 (6.3)	9 (4.8)^§§^	13 (8.1)	9 (6.1)

**Abbreviation**: COVID-NET = Coronavirus Disease 2019–Associated Hospitalization Surveillance Network.

* Data are from a weighted sample of hospitalized infants with completed medical record abstractions. Sample sizes presented are unweighted with weighted percentages.

^†^ Includes infants admitted to a hospital with an admission date during June 20, 2021–August 31, 2022, in 12 states participating in COVID-NET: California, Colorado, Connecticut, Georgia, Michigan, Minnesota, New Mexico, New York, Ohio, Oregon, Tennessee, and Utah. https://www.cdc.gov/mmwr/volumes/69/wr/mm6915e3.htm

^§^ A birth hospitalization was defined as the hospitalization during which the infant was born.

^¶^ Defined as one or more of the following: prematurity, neurologic disorders, congenital heart disease, abnormality of airway, chronic metabolic disease, immunocompromised condition, chronic lung disease of prematurity/bronchopulmonary dysplasia, or asthma/reactive airway disease. Underlying conditions that occurred in fewer than five sampled cases in any stratum are not displayed.

** COVID-19–related signs and symptoms included respiratory conditions (congestion/runny nose, cough, shortness of breath/respiratory distress, upper respiratory infection, wheezing, nausea/vomiting, rash, and seizures) and nonrespiratory conditions (conjunctivitis, diarrhea, fever/chills, apnea, cyanosis, decreased vocalization/stridor, dehydration, hypothermia, inability to eat/poor feeding, and lethargy). Signs and symptoms were abstracted from medical charts and might be incomplete.

^††^ Symptoms that occurred in fewer than five sampled cases in any stratum are not displayed.

^§§^ Relative SE >30%.

## Discussion

Compared with the Delta variant–predominant period, COVID-19–associated hospitalization rates increased among infants aged <6 months during Omicron variant–predominant periods, but the proportion of hospitalized infants aged <6 months with indicators of the most severe illness did not increase. Among infants aged <6 months, the average weekly COVID-19–associated hospitalization rate during the SARS-CoV-2 Omicron BA.2/BA.5 variant–predominant period was similar to that among adults aged 65–74 years and higher than that in other pediatric age groups and younger adults. These findings underscore the continued risk for COVID-19–associated hospitalization among infants aged <6 months, who are ineligible for vaccination.

Multiple factors likely contributed to high COVID-19–associated hospitalization rates among young infants during the Omicron variant–predominant period, including the high infectivity and community transmission of the SARS-CoV-2 Omicron variant and the relatively low threshold for hospitalizing infants for signs and symptoms consistent with COVID-19 (e.g., fever) relative to that in older children (*7*).

High relative hospitalization rates in infants compared with older children, adolescents, and adults aged <65 years during the Omicron BA.2/BA.5 variant–predominant period also reflect lower rates of hospitalization in these other age groups compared with those during the Delta variant–predominant period, as immunity in older age groups has increased through vaccination,^¶¶¶^ previous infection, or both.****

Because infants are more likely to be immunologically naïve, and vaccines are not approved for infants aged <6 months (*8*), maternal COVID-19 vaccination during pregnancy might help to protect young infants. Maternal completion of a 2-dose primary monovalent mRNA COVID-19 vaccination series during pregnancy has been estimated to be 52% effective against COVID-19 hospitalization among infants aged <6 months. This suggests that young infants might receive protection through passive transplacental transfer of maternal antibodies acquired through maternal vaccination. Effectiveness of maternal vaccination in preventing disease in young infants was lower during the early Omicron variant–predominant period (38%) than during the Delta variant–predominant period (80%) (*9*). Surveillance data show that compared with earlier periods, during the recent Omicron variant–predominant periods, a larger proportion of pregnant women received a vaccination series before pregnancy (*9*,*10*). Because of immune evasion when novel variants have emerged and waning immunity as time since the last dose increases, infants born during the Omicron BA.5 variant–predominant period might have had less protection.

The findings in this report are subject to at least five limitations. First, population estimates for infants aged <6 months were not available; therefore, the assumption was that they accounted for one half of infants aged <1 year when calculating rates. This assumption does not account for seasonality in births,^††††^ which was affected by the pandemic; births typically peak in the summer, leading to potential small overestimates of rates during Omicron BA.2/BA.5 variant–predominant periods. Second, it was not possible to account for changes in public health policies and testing and treatment practices over time. Third, maternal vaccination or previous infection, which might confer some immunity to infants, was not assessed. Fourth, periods of variant predominance are based on national data and might not reflect regional differences in circulating variants. Finally, the COVID-NET catchment areas include approximately 10% of the U.S. population; these findings might not be nationally generalizable.

Although these findings do not suggest increased severity of COVID-19 among hospitalized infants, COVID-19–associated hospitalization rates in infants aged <6 months were higher during the Omicron variant–predominant periods (December 2021–August 2022) than they were during previous periods. Maternal vaccination has been shown to provide protection to infants aged <6 months who are currently ineligible for vaccination (*9*), and both CDC and the American College of Obstetricians and Gynecologists recommend COVID-19 vaccination for women who are pregnant, breastfeeding, trying to get pregnant now, or might become pregnant in the future.^§§§§^

COVID-19 hospitalization rates in infants aged <6 months are higher than those of all other age groups except adults aged ≥65 years. To help protect both pregnant women and infants too young to be vaccinated, prevention should focus on ensuring that pregnant women stay up to date on COVID-19 vaccines^¶¶¶¶^ (including receiving a bivalent booster dose)***** and implementing nonpharmaceutical interventions for COVID-19 prevention (*6*) and newborn care.^†††††^

**Summary**

**What is already known about this topic?**

Infants aged <6 months, who are ineligible for vaccination, have high COVID-19–associated hospitalization rates compared with other pediatric age groups.

**What is added by this report?**

Although population-based COVID-19–associated hospitalization rates among infants aged <6 months increased in the Omicron variant–predominant periods compared with the Delta variant–predominant period, indicators of the most severe disease among hospitalized infants aged <6 months did not.

**What are the implications for public health practice?**

Pregnant women should stay up to date with COVID-19 vaccination to help protect themselves and infants too young to be vaccinated. Nonpharmaceutical measures should be used to help protect infants ineligible for vaccination.

## Footnotes

*
https://www.cdc.gov/coronavirus/2019-ncov/covid-data/covid-net/purpose-methods.html

† A variant that accounted for >50% of sequenced isolates in all age groups was considered predominant. https://data.cdc.gov/Laboratory-Surveillance/SARS-CoV-2-Variant-Proportions/jr58-6ysp/data

§ Indicators of severe disease included hospital length of stay, intensive care unit admission, need for respiratory support, and in-hospital death.

¶ COVID-NET includes selected counties in California, Colorado, Connecticut, Georgia, Iowa, Maryland, Michigan, Minnesota, New Mexico, New York, Ohio, Oregon, Tennessee, and Utah. https://www.cdc.gov/mmwr/volumes/69/wr/mm6915e3.htm

** Iowa did not collect demographic or clinical data for the full analysis period and was therefore excluded from all analyses.

†† Signs and symptoms included respiratory conditions (congestion/runny nose, cough, shortness of breath/respiratory distress, upper respiratory infection, wheezing, nausea/vomiting, rash, and seizures) and nonrespiratory conditions (conjunctivitis, diarrhea, fever, apnea, cyanosis, decreased vocalization/stridor, dehydration, hypothermia, inability to eat/poor feeding, and lethargy). Signs and symptoms were abstracted from medical charts and might be incomplete.

§§ Maryland provided demographic data for rate calculations but did not collect clinical data for the full analysis period.

¶¶ During December 2021–January 2022, sites sampled data for 12%–100% of pediatric patients. Random numbers (1–100) were automatically generated and assigned to each patient on entry into the surveillance database to produce random samples of hospitalized patients for medical record abstraction. Percentages were weighted to account for the probability of selection for sampled patients.

*** Rates are calculated using the National Center for Health Statistics’ vintage 2020 bridged-race postcensal population estimates for the counties included in surveillance. https://www.cdc.gov/nchs/nvss/bridged_race.htm

††† 45 C.F.R. part 46.102(l)(2), 21 C.F.R. part 56; 42 U.S.C. Sect. 241(d); 5 U.S.C. Sect. 552a; 44 U.S.C. Sect. 3501 et seq.

§§§ Prematurity was defined as gestational age at birth of <37 weeks.

¶¶¶
https://covid.cdc.gov/covid-data-tracker/#vaccinations_vacc-total-admin-rate-total

****
https://covid.cdc.gov/covid-data-tracker/#nationwide-blood-donor-seroprevalence

††††
https://www.census.gov/library/stories/2021/09/united-states-births-declined-during-the-pandemic.html

§§§§ https://www.cdc.gov/coronavirus/2019-ncov/vaccines/recommendations/pregnancy.html; https://www.acog.org/clinical/clinical-guidance/practice-advisory/articles/2020/12/covid-19-vaccination-considerations-for-obstetric-gynecologic-care

¶¶¶¶
https://www.cdc.gov/coronavirus/2019-ncov/vaccines/recommendations/pregnancy.html

*****
https://www.cdc.gov/vaccines/covid-19/clinical-considerations/interim-considerations-us.html

†††††
https://www.cdc.gov/coronavirus/2019-ncov/if-you-are-sick/pregnancy-breastfeeding.html
