# Erratum: Vol. 71, No. 45

**DOI:** 10.15585/mmwr.mm7150a7

**Published:** 2022-12-16

In the report “COVID-19–Associated Hospitalizations Among U.S. Infants Aged <6 Months — COVID-NET, 13 States, June 2021–August 2022,” on page 1442, the fifth sentence of the first paragraph should have read, “During the Omicron BA.2/BA.5–predominant periods (**March 20**–August 31, 2022), weekly hospitalizations per 100,000 infants aged <6 months increased from a nadir of 2.2 (week ending April 9, 2022) to a peak of 26.0 (week ending July 23, 2022), and the average weekly hospitalization rate among these infants (13.7) was similar to that among adults aged 65–74 years (13.8).” In addition, on page 1443, the last sentence of the fourth paragraph should have read, “The mean weekly hospitalization rate among infants aged <6 months during the Omicron BA.2/BA.5 period (13.7) was less than that of adults aged ≥75 years (39.4), similar to that of adults aged 65–74 years (13.8) and higher than rates in all other pediatric age groups (2.3 and 0.8 for children aged **6 months–4 years and 5–17 years**, respectively) and in adults aged <65 years (**4.6**).”

